# Familial fibrosing frontal alopecia in six sisters^☆^^☆☆^

**DOI:** 10.1016/j.abd.2019.02.009

**Published:** 2019-12-20

**Authors:** Vanessa Barreto Rocha, Mario Cezar Pires, Leticia Arsie Contin

**Affiliations:** Hospital do Servidor Público Municipal de São Paulo, São Paulo, SP, Brazil

*Dear Editor*,

Frontal fibrosing alopecia (FFA) is a progressive cicatricial alopecia that was first described by Kossard in 1994 and has been increasingly reported worldwide in the last decade. Familial cases of FFA have been described since 2008,^1^ but all series include up to three relatives in the same generation or five in two generations.^2^, ^3^ We encountered a group of six sisters presenting clinical and histological diagnosis of FFA in Brazil.

This family consists of eight siblings, two brothers (not examined), and six sisters, who are all committed by this condition (Fig. 1). The sisters’ parents were not consanguineous, and were not examined, they are deceased. The sisters live in the same city; however, they do not live together. All of them are postmenopausal, and none has taken Hormone Replacement Therapy (HRT). The age at FFA onset ranges from 31 years to 62 years while the mean age is 50.7 years. Five have loss of eyebrows and eyelashes, and four have loss of body hair. Lichen Planus Pigmentosus (LPPig) is noted in two sisters and facial papules in three. The diagnosis was made by clinical and tricoscopic characteristics (Fig. 2). They refused to be submitted to biopsy. As a differential diagnosis, it could be considered atrophic pilar keratosis, but none of them presented pilar keratosis.

**Figure 1:**
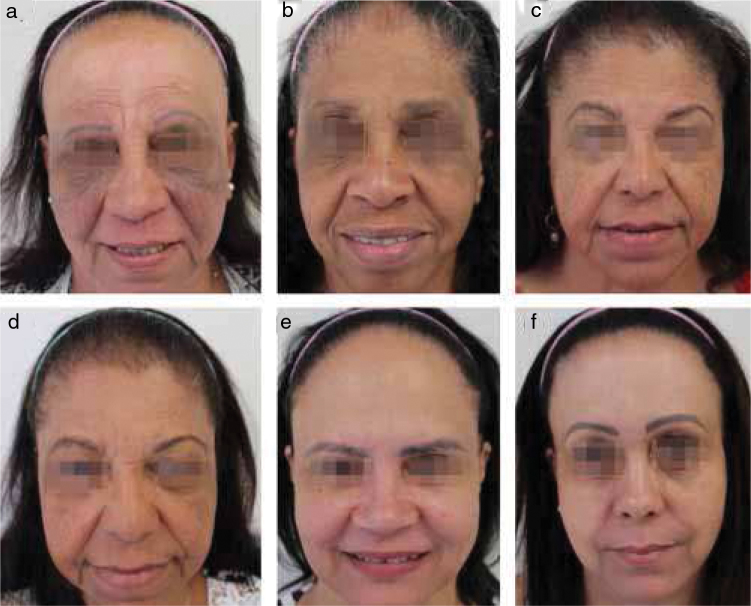
Six sisters with FFA, named by letters, being (a) the oldest and (f) the youngest.

**Figure 2:**
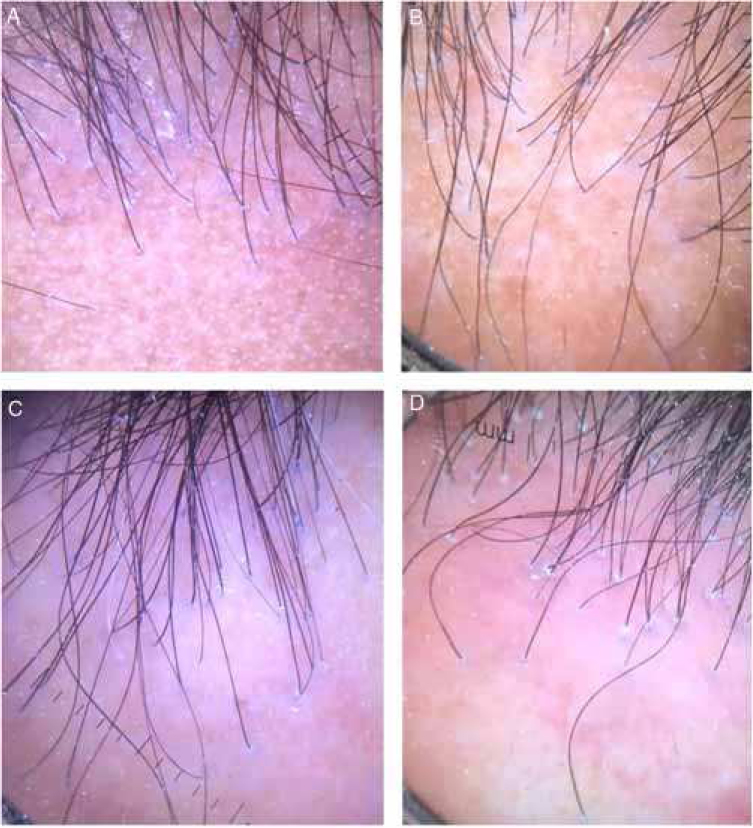
Tricoscopy of the frontal implantation region of patients a, b, e, f, showing absence of vellus and erythema and perifollicular desquamation in all cases, and interfollicular desquamation in C and D.

Associated diseases are Systemic Arterial Hypertension (SAH) and Diabetes Mellitus (DM) in one patient, Deep Vein Thrombosis (DVT) and allergic rhinitis in two other. There are no cases of thyroid dysfunction. Clinical and demographic data are summarized in table 1.

**Table 1:** Clinical and demographic data of the six sisters with frontal fibrosing alopecia.

Patient	Patient age/phototype	Age at diagnosis	Clinical subtype of FFA	LPPig	Eyebrow loss	Eyelashes loss	Body hair loss	Facial papules	Age of menopause/HRT	Comorbidities
1	67y, III	52y	II (diffuse)	Yes	Yes	Yes	Yes	No	55y/no	SAH, DM
2	64y, V	62y	Patchy + II	No	Yes, partial	Yes	No	No	48y/no (hysterectomy)	No
3	62y, IV	60y	II	No	No	Yes	Yes	No	55y/no	No
4	61y, IV	31y	II	No	Partial	Yes	Yes	Yes	48y/no	Allergic rhinitis
5	52y, III	50y	II	No	Yes	No	Yes	Yes	49y/no	DVT
6	51y, III	49y	II	Yes	Yes	Yes	No	Yes	51y/no	No

LPPig, Lichen Planus Pigmentosus; HRT, Hormone Replacement Therapy; SAH, Systemic Arterial Hypertension; DM, Diabetes Mellitus; DVT, Deep Vein Thrombosis.

Clinical patterns of FFA have been described based upon frontal hairline recession as pattern I (linear), pattern II (diffuse), pattern III (pseudo-“fringe sign”)^4^ and patchy pattern.^5^ In our series pattern II is found in all of the patients and patchy pattern in one.

The genetic of FFA is still unknown. Studies show that HLA-DR1 has been implicated in lichen planus and Lassueur–Graham–Little–Piccardi syndrome, but it was not found in FFA.^1^ Familial cases could indicate a genetic mechanism.

Moreover, the FFA epidemic seen nowadays strongly suggests an environmental trigger is also involved. In this series we could not define any substance that could be pointed as this factor, even though they all used sunscreen at some point in their lives.

To our knowledge, this is the largest series with FFA in the same family ever described. Studying these families could help to understand the genetic and the pathogenesis of this intriguing disease.

## Financial support

None declared.

## Author's contribution

Vanessa Barreto Rocha: Approval of the final version of the manuscript; conception and planning of the study; elaboration and writing of the manuscript; collection, analysis, and interpretation of data; critical review of the literature; critical review of the manuscript.

Mario Cezar Pires: Approval of the final version of the manuscript; effective participation in research orientation; critical review of the literature.

Leticia Arsie Contin: Approval of the final version of the manuscript; conception and planning of the study; elaboration and writing of the manuscript; collection, analysis, and interpretation of data; effective participation in research orientation; intellectual participation in propaedeutic and/or therapeutic conduct of the cases studied; critical review of the manuscript.

## Conflicts of interest

None declared.
